# Supplementary material for: Dynamic spatiotemporal determinants modulate GPCR:G protein coupling selectivity and promiscuity
Source: Nat Commun. 2022 Dec 2;13:7428. doi: 10.1038/s41467-022-34055-5 (PMC9718833; doi:10.1038/s41467-022-34055-5)
Supplement: Supplementary file 1 — Supplementary Information [file 41467_2022_34055_MOESM1_ESM.pdf]

Supplementary Table 1: Sequences of the primers

Primer Name	Sequence (5'-3')
S126A-F	CTA CTT CGC GGT GAC TCG GCC CCT G
S126A-R	GGG CCG AGT CAC CGC GAA GTA GCG GTC AAA G
M1_L131F-F1	GAC TCG GCC CTT CAG CTA CCG TGC CAA G
M1_L131F-R1	ACG GTA GCT GAA GGG CCG AGT CAC GGA G
P139K-F	AAG CGC ACA AAA CGC CGG GCA GCT CTG
P139K-R	CTG CCC GGC GTT TTG TGC GCT TGG CAC G
R218Q-F	CAG AGA ACC AGG CAC GGG AGC TGG CAG
R218Q-R	AGC TCC CGT GCC TGG TTC TCT GTC TCC CGG TAG
E221K-F	AGC ACG GAA ACT GGC AGC CCT TCA G
E221K-R	AAG GGC TGC CAG TTT CCG TGC TCG GTT CTC
A424K-F	AAA AAT TCC GGG ACA CCT TTC GCC TGC
A424K-R	CGA AAG GTG TCC CGG AAT TTT TTG TTG CAG AGT GC
Ori-F	CGC TGT AGG TAT CTC AGT TCG GTG TAG GTC GTT CGC TCC
Ori-R	CCT CGC TTG CTG GAT GTG GCT TGA CTC TAT GGA TGT CGC
ultramer_HA-M1-F	TAA ACT TAA GCT TGG TAC CGA GCT CGG ATC CAC CAT GTA CCC ATA CGA CGT CCC AGA CTA CGC CAA CAC TTC AGC CCC ACC TGC TGT
M1-blp1-R	GTA CCG ATG ATG AGG TCA GCA C

DNA sequences for all primers used to verify incorporation of mutations into CHRM1 BRET Biosensors.

Supplementary Figure 1: GPCR:G protein coupling is a dynamic conformational ensemble

For all ensembles shown in figure, the Cα atoms of the GPCR:G protein Ras-domain were aligned to the Cα atoms of ADRB2:Gαs protein (3SN6) to keep the viewing angle consistent across all G protein coupling interactions. **a)** The interaction of Class A Gs-coupled GPCRs with the α5 helix of the Gαs protein. These conformations of the α5 helix from multiple different PDBs (ADORA2A:5G53, ADRB1:7JJO, ADRB2:3SN6, ADRB3:7DH5, CCKAR:7MBX, DRD1:7JVP, GPBAR:7CFM, GPR52:6LI3, CHRM4:7AUE, OX2R:7L1V, PE2R2:7CX2, AVPR2:7KH0) represent a dynamic ensemble of conformational states which sample different intermolecular contacts. The conformation of the GPCR shown is ADRB2 (3SN6). While the α5 helix contributes a large majority of residues identified in intermolecular contacts with GPCRs, several G protein residues outside of the α5 helix form contacts with GPCRs. **b)** The conformational ensemble of the α5 helix of various Gαi proteins (HTR1A:7E2X, HTR1B:6G79, HTR1D:7E32, HTR1E:7E33, ADORA1:6D9H, CHRM2:6OIK, ADRA2B:6K41, CCR5:7O7F, CCR6:6WW2, CNR1:6KPG, CNR2:6PT0, CXCR2:6LFO, DRD2:7JVR, DRD3:7CMV, FPR2:6OMM, NTR1:6OS9, OPRM:6DDF, OPSD:6FUF) interfaced to ADORA1 (6D9H). **c)** The conformational ensemble of the α5 helix of different Gαq proteins (CCKAR:7MBY, HRH1:7DFL, HTR2A:6WHA, CHRM1:6OIJ) interfaced to HTR2A. **d)** The four conformational ensembles of the NTRS1:$G\alpha_{i1}$ complex. The canonical conformations are shown from 6OS9 and 7L0Q, while non-canonical conformations are shown from 6OSA and 7L0S.

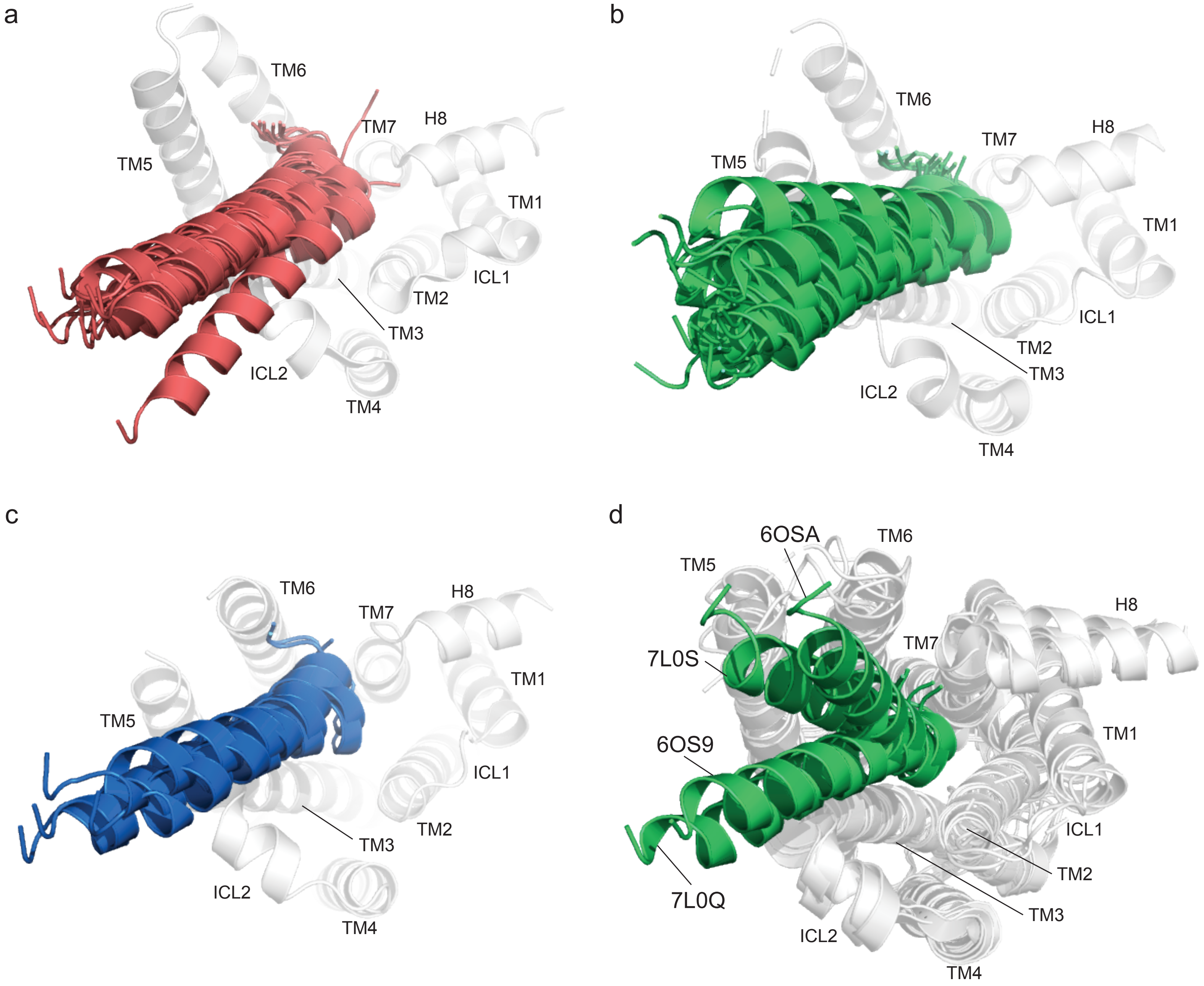
a
TM6
TM7
H8
TM5
TM1
ICL1
TM2
TM3
ICL2
TM4
b
TM6
TM7
H8
TM5
TM1
ICL1
TM2
TM3
ICL2
TM4
c
TM6
TM7
H8
TM1
TM5
ICL1
TM2
TM3
ICL2
TM4
d
6OSA
TM6
TM5
H8
TM7
7L0S
TM1
6OS9
ICL1
TM2
TM3
7L0Q
ICL2
TM4

Supplementary Figure 2: Signatures of subfamily specific and common contacts across the GPCR:G protein interaction

a) Gαs subfamily specific contacts sampled in >20% of snapshots from MD simulations of ADRB2 or ADORA2A with Gs proteins. **b)** Gαi subfamily specific contacts sampled in >20% of snapshots from MD simulations of ADORA1 or HTR1B with Gi proteins. **c)** Gαq subfamily specific contacts sampled in >20% of snapshots from MD simulations of CHRM1 or HTR2A. **d)** weblogo plot of the amino acid frequencies from residue positions found among "common contacts" from the 6 simulated GPCRs. **e)** weblogo plot of the amino acid frequencies from residue positions found among "common contacts" from the 5 simulated G proteins.

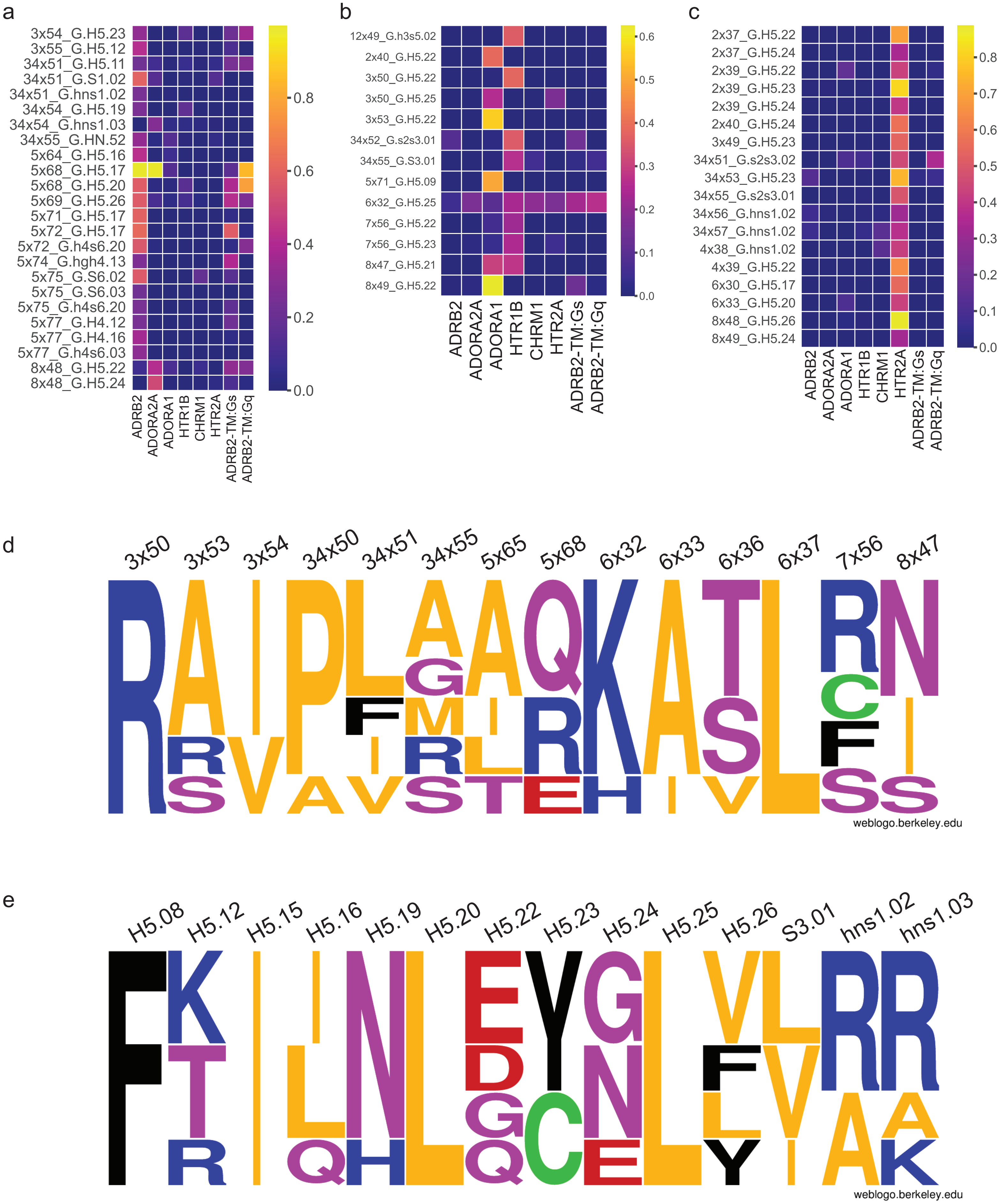

a
3x54_G.H5.23
3x55_G.H5.12
34x51_G.H5.11
34x51_G.S1.02
34x51_G.hns1.02
34x54_G.H5.19
34x54_G.hns1.03
34x55_G.HN.52
5x64_G.H5.16
5x68_G.H5.17
5x68_G.H5.20
5x69_G.H5.26
5x71_G.H5.17
5x72_G.H5.17
5x72_G.h4s6.20
5x74_G.hgh4.13
5x75_G.S6.02
5x75_G.S6.03
5x75_G.h4s6.20
5x77_G.H4.12
5x77_G.H4.16
5x77_G.h4s6.03
8x48_G.H5.22
8x48_G.H5.24
ADRB2
ADORA2A
ADORA1
HTR1B
CHRM1
HTR2A
ADRB2-TM:Gs
ADRB2-TM:Gq
0.8
0.6
0.4
0.2
0.0
b
12x49_G.h3s5.02
2x40_G.H5.22
3x50_G.H5.22
3x50_G.H5.25
3x53_G.H5.22
34x52_G.s2s3.01
34x55_G.S3.01
5x71_G.H5.09
6x32_G.H5.25
7x56_G.H5.22
7x56_G.H5.23
8x47_G.H5.21
8x49_G.H5.22
ADRB2
ADORA2A
ADORA1
HTR1B
CHRM1
HTR2A
ADRB2-TM:Gs
ADRB2-TM:Gq
0.6
0.5
0.4
0.3
0.2
0.1
0.0
c
2x37_G.H5.22
2x37_G.H5.24
2x39_G.H5.22
2x39_G.H5.23
2x39_G.H5.24
2x40_G.H5.24
3x49_G.H5.23
34x51_G.s2s3.02
34x53_G.H5.23
34x55_G.s2s3.01
34x56_G.hns1.02
34x57_G.hns1.02
4x38_G.hns1.02
4x39_G.H5.22
6x30_G.H5.17
6x33_G.H5.20
8x48_G.H5.26
8x49_G.H5.24
ADRB2
ADORA2A
ADORA1
HTR1B
CHRM1
HTR2A
ADRB2-TM:Gs
ADRB2-TM:Gq
0.8
0.7
0.6
0.5
0.4
0.3
0.2
0.1
0.0
d
3x50
3x53
3x54
34x50
34x51
34x55
5x65
5x68
6x32
6x33
6x36
6x37
7x56
8x47
weblogo.berkeley.edu
e
H5.08
H5.12
H5.15
H5.16
H5.19
H5.20
H5.22
H5.23
H5.24
H5.25
H5.26
S3.01
hns1.02
hns1.03
weblogo.berkeley.edu

Supplementary Figure 3: GPCR:G protein coupling is mediated by a core set of common contacts

a) The cell surface expression of CHRM1 WT and mutant BRET biosensors was measured in transfected HEK293T cells using ELISA with anti-HA HRP (n=3 biologically independent samples, data are presented as mean ±SD for each concentration). **b)** Residues identified in the LDA model that distinguish binding to different G protein subfamilies were assessed from a population genetics perspective to determine the level of natural variation at these selectivity-conferring positions compared to the background variation at GPCR residue positions. The bar-graph displays the mean variant allele frequency per residue of the collective GPCR residues among LDA-determined contacts for each receptor compared to the mean variant allele frequency per residue in the remaining residues of each receptor. The y-axis is plotted in reverse logarithmic order, with taller bars representing lower overall allele frequencies. Source data are provided as Supplementary Data 8. **c)** The GPCR residues found within the Gs-coupled LDA-determined contacts are shown on the x-axis. The barplot displays the average "Normalized Activity" (± SEM) of each position when mutated to each of 19 alternate amino acids in the ADRB2 and stimulated with 625nM isoproterenol, as quantified in Jones et al. 2020 eLife. The dashed horizontal line represents the global average "Normalized Activity" of 1.75 units, calculated from the pooled activity of all variants across all conditions of isoproterenol stimulation. **d)** DRD1:Gs contacts overlapped highly with Gs simulations from the LDA model, with contacts predicted to be in the Gs class with a score of 87.73%. **e)** CNR1:Gi contacts show overlap with both Gi and Gq contacts from the LDA model, with Gi class prediction at 41.39%. **f)** HRH1:Gq contacts also show overlap with both Gq and Gi class contacts, with overall prediction for Gq class at 21.61%. **g)** The contacts considered "common" or subfamily "specific" were collectively used to calculate the non-bond interaction energy (coulombic and van der Waals forces) from MD simulations using the "gmx_energy" utility of Gromacs. Data are represented as the summed average energies for pairwise contacts from the common and G protein specific interactions identified for each GPCR:G protein pair, calculated from the terminal 100ns of each replicated MD simulation. Data are presented as mean ± SD from 5 replicate MD trajectories. **h)** The subfamily specific contacts are sampled at a lower average frequency than the common contacts for each GPCR:G protein complex analyzed. We further assessed whether the entire network of contacts (those not among the common contacts or subfamily specific contacts) sampled in each G

protein class was under sampled compared to the common contacts. The “class_sampled” frequencies contain all contacts sampled for at least 20% frequency in one or both simulations for each class.

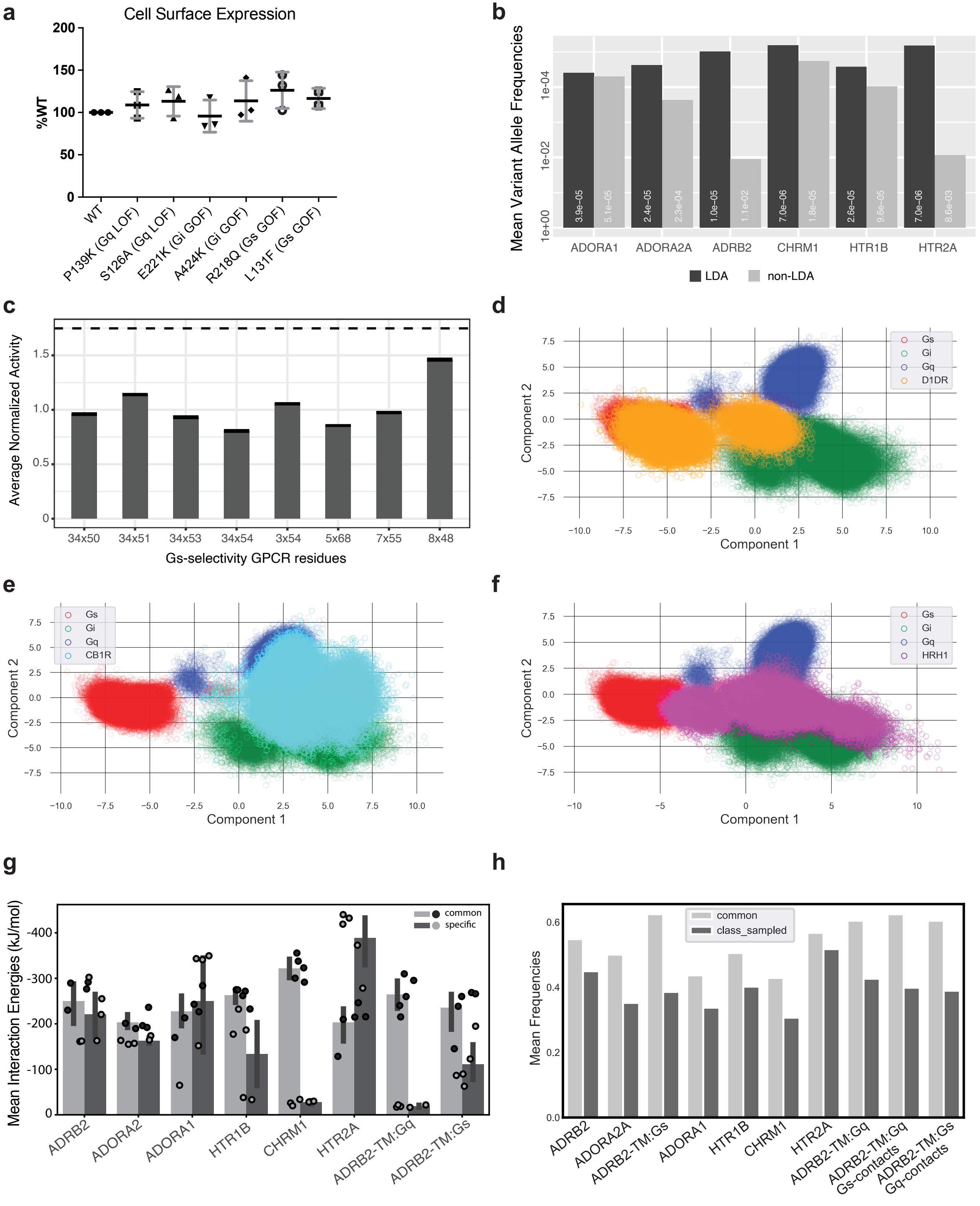
a
Cell Surface Expression
%WT
200
150
100
50
0
WT
P139K (Gq LOF)
S126A (Gq LOF)
E221K (Gi GOF)
A424K (Gi GOF)
R218Q (Gs GOF)
L131F (Gs GOF)
b
Mean Variant Allele Frequencies
1e-04
1e-02
1e+00
3.9e-05
5.1e-05
2.4e-05
2.3e-04
1.0e-05
1.1e-02
7.0e-06
1.8e-05
2.6e-05
9.6e-05
7.0e-06
8.6e-03
ADORA1
ADORA2A
ADRB2
CHRM1
HTR1B
HTR2A
LDA
non-LDA
c
Average Normalized Activity
0
0.5
1.0
1.5
34x50
34x51
34x53
34x54
3x54
5x68
7x55
8x48
Gs-selectivity GPCR residues
d
Gs
Gi
Gq
D1DR
Component 2
Component 1
e
Gs
Gi
Gq
CB1R
Component 2
Component 1
f
Gs
Gi
Gq
HRH1
Component 2
Component 1
g
Mean Interaction Energies (kJ/mol)
common
specific
ADRB2
ADORA2
ADORA1
HTR1B
CHRM1
HTR2A
ADRB2-TM:Gq
ADRB2-TM:Gs
h
Mean Frequencies
common
class_sampled
ADRB2
ADORA2A
ADRB2-TM:Gs
ADORA1
HTR1B
CHRM1
HTR2A
ADRB2-TM:Gq
ADRB2-TM:Gq Gs-contacts
ADRB2-TM:Gs Gq-contacts

Supplementary Figure 4: Building a Linear Discriminant Analysis classifier for GPCR:G protein selectivity

a) Prior to using the LDA classifier for feature selection, we trained the LDA model using 80% of the total fingerprint dataset. The deconvoluted 2-dimensional space is plotted such that fingerprints from the Gαs coupled interactions are shown in red, Gαi fingerprints are plotted in green, and Gαq fingerprints are shown in blue. **b)** The 20% of fingerprints used as holdout data were tested based on the training LDA model. The holdout data were plotted onto the LDA space such that Gαs coupled interactions are shown with orange “x”, Gαi fingerprints are plotted in cyan “x”, and Gαq fingerprints are shown in purple “x”. The testing holdout validation performed at 99.64% accuracy.

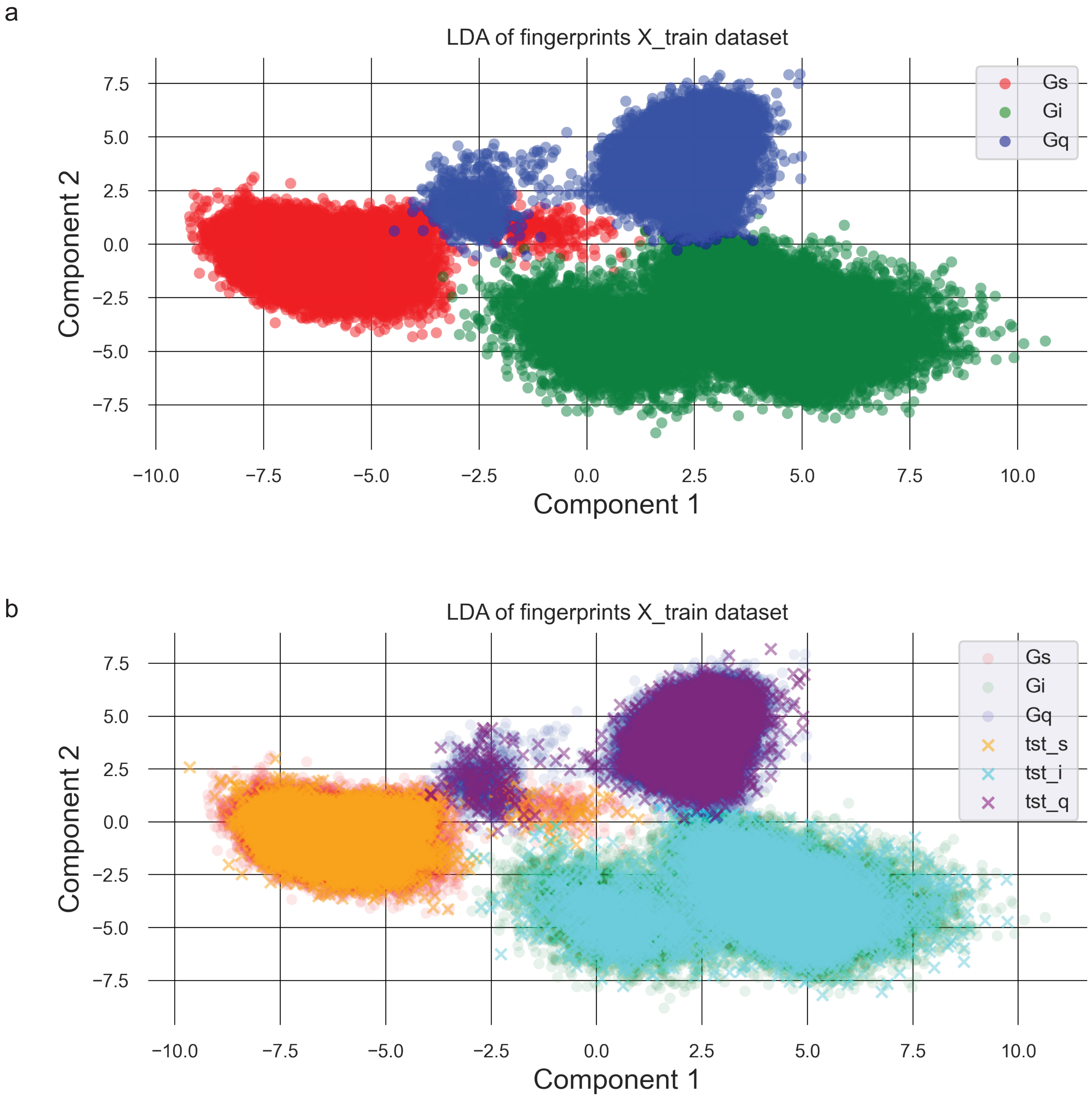

a
LDA of fingerprints X_train dataset
Component 2
Component 1
Gs
Gi
Gq
b
LDA of fingerprints X_train dataset
Component 2
Component 1
Gs
Gi
Gq
tst_s
tst_i
tst_q
