# Supplementary material for: Dynamic spatiotemporal determinants modulate GPCR:G protein coupling selectivity and promiscuity
Source: Nat Commun. 2022 Dec 2;13:7428. doi: 10.1038/s41467-022-34055-5 (PMC9718833; doi:10.1038/s41467-022-34055-5)
Supplement: Supplementary file 3 — Description of Additional Supplementary Files [file 41467_2022_34055_MOESM3_ESM.pdf]

**Description of Additional Supplementary Files**

File Name: Supplementary Data 1:

Description: “SourceDataS1_Gprotein_Subfamily_promiscuity_index.xlsx” GPCR:G protein coupling data was aggregated from those available on GPCRdb.org (Inoue et al11 and IUPHAR database) and the Avet et al12 study to identify consensus G protein coupling information across multiple datasets. We assigned higher weight to the couplings identified in the Avet study, as these coupling interactions were measured using WT GPCR and WT G protein sequences in the FRET Biosensors, whereas the Inoue study used a chimeric Gαq protein sensor which only incorporates the terminal 6 amino acids of each Gα protein.

File Name: Supplementary Data 2

Description: “SourceDataS2_GPCR_Gprotein_contact_fingerprints_forLDA.txt” Intermolecular contacts were identified from each from of each trajectory in the 6 GPCR:G protein complexes used to build the LDA model. We used one-hot encoding to generate a binary “fingerprint” of all intermolecular interactions in each frame of the trajectories.

File Name: Supplementary Data 3

Description: “SourceDataS3_LDA_Simulation_Contact_Frequencies.xlsx” For each GPCR:G protein complex simulated via a MD trajectory, we computed the frequency of formation for each contact identified in the entire dataset.

File Name: Supplementary Data 4

Description: “SourceDataS4_frame_coordinates_LDA_model.csv” After generating the LDA model, we transformed each fingerprint from Supplementary Data 2 into the resulting LDA deconvoluted space, made up of the vectors “Component 1” and “Component 2.”

File Name: Supplementary Data 5

Description: “SuppDataS5_weighted_coefficients_LDA_model.csv” After performing LDA, we obtained the coefficients and mean frequencies for each contact within the Gs, Gi, and Gq coupled interactions. We created a new metric by taking the product of the coefficient values and the mean values for each contact, for each class (Gs, Gi, and Gq).

File Name: Supplementary Data 6

Description: “SuppDataS6_LDA_Spatiotemporal_Code_weights.xlsx” The top-10 weighted coefficients, from Supplementary Data 5, for each class. We also included whether the contact was found in one of the pdbs for the starting structures of the GPCR:G protein complex modeled for the given class.

File Name: Supplementary Data 7

Description: “SuppDataS7_covariance_matrix_LDA_model.csv” Weighted within-class covariance matrix from Gs, Gi, and Gq groups in the LDA model.

File Name: Supplementary Data 8

Description: “SourceDataS8_gnomAD_frequencies.xlsx” We obtained the frequencies of all missense mutations identified in the gnomAD database for the GPCRs used in this study.

File Name: Supplementary Data 9

Description: “fingerprint_python_scripts.zip” These scripts were used to generate binary fingerprints for each MD trajectory used in the study.
