# Supplementary material for: Dynamic spatiotemporal determinants modulate GPCR:G protein coupling selectivity and promiscuity
Source: Nat Commun. 2022 Dec 2;13:7428. doi: 10.1038/s41467-022-34055-5 (PMC9718833; doi:10.1038/s41467-022-34055-5)
Supplement: Supplementary file 13 — Reporting Summary [file 41467_2022_34055_MOESM13_ESM.pdf]

nature portfolio

Corresponding author(s): Nagarajan Vaidehi, Madan Babu, Manbir Sandhu

Last updated by author(s): 2022/09/27

Reporting Summary

Nature Portfolio wishes to improve the reproducibility of the work that we publish. This form provides structure for consistency and transparency in reporting. For further information on Nature Portfolio policies, see our Editorial Policies and the Editorial Policy Checklist.

Statistics

For all statistical analyses, confirm that the following items are present in the figure legend, table legend, main text, or Methods section.

n/a	Confirmed	
☐	☒	The exact sample size (*n*) for each experimental group/condition, given as a discrete number and unit of measurement
☐	☒	A statement on whether measurements were taken from distinct samples or whether the same sample was measured repeatedly
☐	☒	The statistical test(s) used AND whether they are one- or two-sided<br>*Only common tests should be described solely by name; describe more complex techniques in the Methods section.*
☒	☐	A description of all covariates tested
☒	☐	A description of any assumptions or corrections, such as tests of normality and adjustment for multiple comparisons
☐	☒	A full description of the statistical parameters including central tendency (e.g. means) or other basic estimates (e.g. regression coefficient) AND variation (e.g. standard deviation) or associated estimates of uncertainty (e.g. confidence intervals)
☐	☒	For null hypothesis testing, the test statistic (e.g. *F*, *t*, *r*) with confidence intervals, effect sizes, degrees of freedom and *P* value noted<br>*Give P values as exact values whenever suitable.*
☒	☐	For Bayesian analysis, information on the choice of priors and Markov chain Monte Carlo settings
☒	☐	For hierarchical and complex designs, identification of the appropriate level for tests and full reporting of outcomes
☒	☐	Estimates of effect sizes (e.g. Cohen's *d*, Pearson's *r*), indicating how they were calculated

Our web collection on statistics for biologists contains articles on many of the points above.

Software and code

Policy information about availability of computer code

Data collection	Gen5 v3.11 Data Analysis software, GROMACS 2022
Data analysis	Graph Pad Prism 6, Python 3.7, pandas 1.4.3, scipy 1.8.1, Scikit-learn 1.1.1, Matplotlib 3.5.1, seaborn 0.11.2, ggplot2_3.3.6, R 4.1.2, Jupyter-notebook 5.0

For manuscripts utilizing custom algorithms or software that are central to the research but not yet described in published literature, software must be made available to editors and reviewers. We strongly encourage code deposition in a community repository (e.g. GitHub). See the Nature Portfolio guidelines for submitting code & software for further information.

Data

Policy information about availability of data

All manuscripts must include a data availability statement. This statement should provide the following information, where applicable:

- Accession codes, unique identifiers, or web links for publicly available datasets
- A description of any restrictions on data availability
- For clinical datasets or third party data, please ensure that the statement adheres to our policy

All data and analysis scripts used during the current study are included in this published article (and its supplementary information files). The molecular dynamics simulations datasets generated for the current study ARE available in the GPCRMD.org repository with the following dynamics IDs: 1190, 1200, 1201, 1203, 1204, 1207, 1209, 1212, 1215, 1214. The structures used for molecular dynamics are found in the Protein Data Bank repository under the following PDB IDs: 3SN6, 6GDG, 6D9H, 6G79, 6OIJ, 6WHA, 7JVP, 6N4B, 7DFL.

Field-specific reporting

Please select the one below that is the best fit for your research. If you are not sure, read the appropriate sections before making your selection.

☒ Life sciences ☐ Behavioural & social sciences ☐ Ecological, evolutionary & environmental sciences

For a reference copy of the document with all sections, see nature.com/documents/nr-reporting-summary-flat.pdf

Life sciences study design

All studies must disclose on these points even when the disclosure is negative.

Sample size	No statistical methods were used to predetermine sample sizes. All experiments were done at least three independent times, and sample sizes are similar to those reported in the literature for with these kind of assays (Namkung et al., Sci Signal. 2018 Dec 4;11(559):eaat1631; Luttrell et al., Sci Signal. 2018 Sep 25;11(549):eaat7650; Beautrait et al., Nat Commun. 2017 Apr 18;8:15054 and Namkung et al., Nat Commun. 2016 Jul 11;7:12178) .
Data exclusions	No data were excluded from our analyses.
Replication	We repeated all experiments using at least three biological replicates (unless stated otherwise) over distinct independent experiments. The number of biological replicates and independent experiments are reported in the figure legends.
Randomization	No randomization was undertaken.
Blinding	Blinding was not applied.

Reporting for specific materials, systems and methods

We require information from authors about some types of materials, experimental systems and methods used in many studies. Here, indicate whether each material, system or method listed is relevant to your study. If you are not sure if a list item applies to your research, read the appropriate section before selecting a response.

Materials & experimental systems

n/a	Involved in the study	
☐	☒	Antibodies
☐	☒	Eukaryotic cell lines
☒	☐	Palaeontology and archaeology
☒	☐	Animals and other organisms
☒	☐	Human research participants
☒	☐	Clinical data
☒	☐	Dual use research of concern

Methods

n/a	Involved in the study	
☒	☐	ChIP-seq
☒	☐	Flow cytometry
☒	☐	MRI-based neuroimaging

Antibodies

Antibodies used	anti-HA-Peroxidase 3F10 (Cat. No. 12 013 819 001, lot #12013819001) was from Sigma Aldrich
Validation	HA-Peroxidase 3F10 antibody, was validated by vendor using the the sequence peptide YPYDVPDYA and GST-HA; https://www.sigmaaldrich.com/deepweb/assets/sigmaaldrich/product/documents/760/007/12013819001bul.pdf

Eukaryotic cell lines

Policy information about cell lines

Cell line source(s)	HEK293T cells were form American Type Culture Collection (ATCC)
Authentication	Cells were not authenticated
Mycoplasma contamination	Cells were tested on a regular basis for mycoplasma contamination, and free of contaminations.
Commonly misidentified lines (See ICLAC register)	HEK293 cells
